# Constraints on infants’ ability to extract non-adjacent dependencies from vowels and consonants

**DOI:** 10.1016/j.dcn.2022.101149

**Published:** 2022-08-31

**Authors:** Ivonne Weyers, Claudia Männel, Jutta L. Mueller

**Affiliations:** a Department of Linguistics, University of Vienna, Sensengasse 3a, 1090 Vienna, Austria; b Institute of Cognitive Science, University of Osnabrück, 49069 Osnabrück, Germany; c Department of Audiology and Phoniatrics, Charité - Universitätsmedizin Berlin, Augustenburger Platz 1, 13353 Berlin, Germany; d Department of Neuropsychology, Max Planck Institute for Human Cognitive and Brain Sciences, Stephanstr. 1A, 04103 Leipzig, Germany

**Keywords:** Vowels, Consonants, Non-adjacent dependency, EEG, Artificial language, Language acquisition

## Abstract

Language acquisition requires infants’ ability to track dependencies between distant speech elements. Infants as young as 3 months have been shown to successfully identify such non-adjacent dependencies between syllables, and this ability has been related to the maturity of infants’ pitch processing. The present study tested whether 8- to 10-month-old infants (*N* = 68) can also learn dependencies at smaller segmental levels and whether the relation between dependency and pitch processing extends to other auditory features. Infants heard either syllable sequences encoding an item-specific dependency between non-adjacent vowels or between consonants. These frequent standard sequences were interspersed with infrequent intensity deviants and dependency deviants, which violated the non-adjacent relationship. Both vowel and consonant groups showed electrophysiological evidence for detection of the intensity manipulation. However, evidence for dependency learning was only found for infants hearing the dependencies across vowels, not consonants, and only in a subgroup of infants who had an above-average language score in a behavioral test. In a correlation analysis, we found no relation between intensity and dependency processing. We conclude that item-specific, segment-based non-adjacent dependencies are not easily learned by infants and if so, vowels are more accessible to the task, but only to infants who display advanced language skills.

## 1 Introduction

Speech is a spectrally and temporally complex auditory signal composed of structured sequences of sounds. In becoming competent language users, infants need to efficiently decode this signal by identifying its subcomponents, namely words, phrases, and sentences, as well as their structural relation to one another. Structural relations can exist between non-neighboring elements (e.g., ‘The girl *was* sing*ing.’*) and require the listener to track their dependency across intervening speech elements (e.g., *was* and *-ing* across sing*-*). These so-called non-adjacent dependencies (NADs) are an integral part of language and appear, for instance, in the form of subject-verb agreement, case or tense marking. The present study investigates stimulus-related factors contributing to NAD learning in 8- to 10-month-old infants. As previous work has focused on infant learning of NADs between syllables (e.g., Mueller et al., 2012), we here focus on this ability based on segmental information, namely dependencies between consonants and vowels. This is relevant because early learning of NADs has been hypothesized to rely strongly on phonetic surface-level forms before more abstract, categorical relations are established later on (Culbertson et al., 2016, Mueller et al., 2020). In addition, we test for the impact of two interindividual factors that have previously been identified as predictors of learning outcome in similar learning tasks, that is auditory perceptual abilities and general language development (e.g., Frost et al., 2020; Mueller et al., 2012).

### 1.1 Non-adjacent dependency learning in early infancy

The ability to learn dependencies between non-adjacent speech elements has been shown in infants already in the first year of life (Kovács, 2014, Marcus et al., 1999, Mueller et al., 2012). For example, in an EEG experiment, Mueller et al. (2012) exposed 3- to 4-month-olds to trisyllabic sequences encoding an NAD between specific syllable frames (e.g., *le*ro*bu*, *le*ka*bu*, *fi*me*to*, *fi*su*to*). The authors found an early Mismatch Response (MMR) for interspersed deviant sequences violating the dependency (e.g., *le*su**to**, *fi*wa**bu**), indexing successful learning and dissociation. Some of the above studies (Kovács, 2014, Marcus et al., 1999) have used repetition-based structures (ABA), e.g., *wo*fe*wo*, z*a*mo*za*, *du*ba*du*, *lo*vu*lo*, to investigate NAD learning in infancy because, similarly to natural syntactic structures, they require generalization beyond a given set of exemplars. It has been suggested, however, that repetitions may be captured by an automatic process of the auditory perceptual system or even a domain-general “repetition detector” (Gervain et al., 2008), which would make them less suitable for the comparison with natural language NADs. Others studies, like Mueller et al. (2012), have instead used item-specific dependencies (AXB) because they arguably better reflect particularly early stages of structural learning in natural language, which may initially be based on phonological surface level forms (e.g., ‘when *was* then *-ing*’ in ‘the girl *was* sing*ing*’) before they develop into more abstract, categorical representations (e.g., ‘when past progressive, form of be then morphological tense marker on main verb’) (Culbertson et al., 2016). The present study investigates item-specific dependencies, as we are interested in the early stages of NAD learning in young infants and the linguistic cues relevant to this process.

### 1.2 Consonants and vowels- different roles in language processing?

Based on substantial research on the potentially different roles of consonants and vowels in language processing, it has been proposed that consonants are more important for lexically-related processes than vowels (for a review, cf. Nazzi and Cutler, 2019). Specifically, adult listeners rely on consonantal rather than vocalic information for the computation of conditional co-occurrence statistics (transitional probabilities) during word segmentation (Bonatti et al., 2005, Mehler et al., 2006, Newport and Aslin, 2004, Peña et al., 2002, Toro et al., 2008) as well as for word identification/lexical selection (Cutler et al., 2000, Delle Luche et al., 2014, Marks et al., 2002, Van Ooijen, 1996), and lexical encoding of new words (Creel et al., 2006, Escudero et al., 2016, Havy et al., 2014). In contrast to consonants, vowels have been proposed to play a preferential role in structural learning. Specifically, because vowels carry prosodic information within larger units, they can provide cues to morpho-syntactic structure, that is, at the local word-level regarding language-specific syllable structure, and at the non-local, phrasal level for phonological phrase structure and syntactic constituency (Bonatti et al., 2005, Nespor et al., 2003). Yet, evidence for the particular role of vowels in language processing remains less palpable than for the role of consonants and stems mainly from studies using (non-adjacent) repetition-rules, which both adults (Monte-Ordoño and Toro, 2017, Toro et al., 2008) and infants (Hochmann et al., 2011, Pons and Toro, 2010) learn tentatively better when they are encoded by vowels (e.g., d*a*l*a*, f*o*d*o*) rather than consonants (e.g., *l*u*l*a, *d*a*d*o).

The role of consonants and vowels in item-specific NAD learning has rarely been investigated. It is essential to our understanding of the learning and representation of NADs, however, to understand which parts or units of the input may serve as potential cues to the dependency relationship during early stages of learning. In other words, the present study does not investigate a specific type of NAD in natural language that exclusively consists of either vowels or consonants, but rather more generally addresses the overall salience and role of consonants and vowels in the process of NAD learning. Based on the proposed distinct roles of consonants and vowels in language processing, one might expect a differential involvement of the two segments in item-specific NAD learning as well. More specifically, it may be that vowels are perceived as more reliable cues to NAD relationships, which would extend the proposed vowel advantage in structural learning beyond the repetition-rule context. Newport and Aslin (2004) aimed to examine consonant and vowel-based NAD learning, but admit that the fixed segmental frames (*p*x*g*x*t*x and x*a*x*u*x*e*) they used for their stimuli rather constituted adjacent than non-adjacent dependencies at the segmental level. A first study on NAD learning in adults that directly compared syllables, consonants and vowels as NAD-encoding units showed a clear advantage of the syllable and a tentative advantage of consonants over vowels (Weyers and Mueller, 2022).

Whether this pattern also holds for early development remains an open question and different outcomes are conceivable. First, regarding the comparison of syllables and segmental units (vowels and consonants), segments may play a more important role in early infancy compared to later stages of development. There are some studies that report on the syllable as relevant perceptual unit from early on (e.g., Bertoncini and Mehler, 1981; Räsänen et al., 2018), yet other studies found segmental rather than syllabic information to be ultimately processed and memorized. For example, Benavides-Varela et al. (2012) demonstrated that vowel information is memorized when newborns are presented with simple word forms. Moreover, Gennari et al. (2021) provided evidence that 3-month-olds encode consonants’ relevant articulatory features, which are then combined into a phoneme representation in a second step. Yet, the authors found no evidence for syllable-related neural representations. Second, regarding the comparison of consonants and vowels, consonants have been argued to be more important for the identification of lexical units (e.g., Havy et al., 2014), but it is currently unclear at which age this functional specificity emerges. In syllable-timed languages, such as French and Italian, infants initially seem to show a vowel bias (V-bias) for word identification, which develops into a consonantal bias (C-bias) between 8 and 11 months (e.g., Nishibayashi and Nazzi, 2016; Von Holzen et al., 2018; Von Holzen and Nazzi, 2020); however, findings from stress-timed languages, such as Dutch or English, are less conclusive (e.g., Mani and Plunkett, 2007; Swingley, 2005). Third, NAD learning itself has been shown to undergo fundamental changes during development. Evidence is accumulating that infants younger than two years learn such regularities automatically and relatively unaffected by higher-order mechanisms such as cognitive control, and sometimes even more successfully than adults, especially in passive experimental setups (Mueller et al., 2012). In contrast, older children and adults’ learning of NADs more strongly depends on controlled cognitive processes (Mueller et al., 2019, Paul et al., 2021).

### 1.3 Interindividual differences in non-adjacent dependency learning during development

Language learning experiments often evidence large variability, explained by interindividual differences in perceptual or cognitive abilities, input-related variables, the learner’s sex, or risk factors for language disorders (for a review, see Kidd et al., 2018). A large number of studies have illustrated that individual variation in early auditory abilities predicts later language development (e.g., Chonchaiya et al., 2013; Cristia et al., 2014), and specifically impacts on the processing and learning of structural dependencies (Mueller et al., 2012). Auditory features for which such a relationship has been demonstrated comprise pitch (e.g., Grube et al., 2012; Mueller et al., 2012) and duration (Grube et al., 2012). These findings strongly suggest a link between auditory processing and language acquisition and processing, yet it remains to be shown whether this holds mainly for language-related auditory features (e.g., pitch) or extends to more general auditory features (e.g., intensity). Individual differences in speech-related learning processes have further been found to correlate with other linguistic skills throughout development. Several studies have, for instance, provided evidence for a relation between statistical learning ability (including word segmentation) and children’s concurrent or later vocabulary size (e.g., Frost et al., 2020; Junge et al., 2012), syntactic processing (Kidd and Arciuli, 2016), and literacy skills (Spencer et al., 2015). Together these findings suggest that both differences in perceptual ability and linguistic skills may be explanatory variables for interindividual differences in NAD learning.

### 1.4 The present study

In the present study (pre-registered at OSF: Weyers et al., 2020), we tested whether infants’ ability to learn item-specific NADs previously shown for syllables extends to smaller segments, namely consonants and vowels. By means of event-related potentials, we tested whether infants between 8 and 10 months of age would be able to learn AXB dependencies between specific consonants (e.g., *b*emu*p*a, *b*asu*p*i) or vowels (e.g., g*o*s*ä*m*u*, l*o*was*u*). We chose this age group since both neurophysiological (e.g., Mueller et al., 2012) and behavioral evidence (Marcus et al., 1999) from artificial grammar learning experiments suggests that infants are sensitive to NADs by 8–10 months. Regarding the previously reported relation between basic auditory and NAD learning abilities (Mueller et al., 2012), we further tested whether infants’ intensity processing would be correlated with NAD learning ability. We chose intensity as the manipulated auditory feature because it is an essential feature of the language input, but in contrast to pitch, it is not directly relevant for phoneme discrimination (Trainor and Adams, 2000, Trainor and Desjardins, 2002). Finding such a correlation would suggest a predictive value of auditory perceptual abilities per se for linguistic processing abilities. As planned but not pre-registered, we additionally administered a development test and in an exploratory analysis examined infants’ linguistic skills and their relation to individual differences in NAD learning.

For our ERP study on NAD learning in 8- to 10-month-old infants, we hypothesized that infants would show evidence of NAD learning for vowel-based dependencies and only to a lesser degree (e.g., later onset or smaller amplitude) for consonant-based dependencies, similar to studies with (non-adjacent) repetition-rules (Hochmann et al., 2011, Pons and Toro, 2010). Since findings from previous similar studies with infants varied substantially with regard to polarity, timing and size of the reported ERP effects (e.g., Mueller et al., 2009; Mueller et al., 2019; Winkler et al., 2018), we did not formulate any specific hypotheses with regard to expected characteristics of possible effects. Further, we hypothesized that infants would detect the syllable-based intensity manipulation, as neonates (Sambeth et al., 2009, Tarquinio et al., 1990) have been reported to already detect even small intensity changes in both speech and non-speech stimuli. Given previous pitch-related findings by Mueller et al. (2012), we expected a comparable association between intensity processing and NAD learning. For participants’ assessment of linguistic development (via a general development test), we expected our exploratory analysis to show that individual differences in general linguistic skills would be related to individual differences in NAD learning (see Frost et al., 2020).

## 2 Materials and methods

### 2.1 Participants

The study adheres to the guidelines of the Declaration of Helsinki (2008) and was approved by the Ethics Committee of the University of Osnabrück. Infants’ caregivers gave written informed consent before participation in the experiment. All tested infants had normal hearing, no history of neurological condition and grew up monolingually German or multilingually with German being one of the languages of exposure. Based on previous research with infants of similar ages using EEG and an oddball paradigm (e.g., Männel and Friederici, 2009), we aimed for a minimum of 35 participants entering the final analysis per experimental group, that is 70 infants in the final sample. In total, 91 infants took part in the study. In order to be included in the final data set, a participant had to provide at least 25 % artifact-free trials in each condition (i.e. 48 out of 192 included standard trials [see below] and 16 out of 64 trials in each deviant condition). Twenty-three participants had to be excluded because of high artifact rate in the measured EEG or early termination of the experiment (e.g., due to crying). Of the 68 remaining participants, 35 (18 female) had been pseudo-randomly (in the order of participation) assigned to the vowel group and 33 (14 female) to the consonant group. Due to lockdown constraints put in place because of the COVID-19 pandemic, data collection was unexpectedly cut short and we were unable to meet the intended minimum of 35 participants for the consonant group. Participants were all between 8 and 10 months old and did not significantly differ (p > .05) between groups with regard to age, gestational age and birth weight, as tested with Welch two-samples *t*-tests (cf. Table 1).

**Table 1:** Overview of participant groups of the vowel and the consonant experiments.

	*Vowel group*	*Consonant group*
Participants (N)	35	33
Mean age (days)	280.66 (SD = 17.61)	281.42 (SD = 18.84)
Gestational age (days)	279.03 (SD = 15.66)	276.25 (SD = 14.71)
Birth weight (g)	3414.91 (SD = 459.71)	3563.81 (SD = 499.93)
Sex	18 female 17 male	14 female 18 male
Mean no. trials standard (of 192)	98.69 (SD = 29.97)	76.3 (SD = 23.79)
Mean no. trials dependency deviant (of 64)	31.26 (SD = 11.29)	25.76 (SD = 7.95)
Mean no. trials intensity deviant (of 64)	32.91 (SD = 10.55)	25.94 (SD = 8.6)
Excluded participants (n)	10	13

### 2.2 Stimuli

**Fig. 1:**
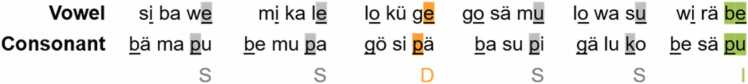
Exemplary series of standard and deviant stimuli by experimental group, dependent elements underlined. S = standard (highlighted in grey), D = dependency deviant (highlighted in orange), I = intensity deviant (highlighted in green). Vowel standards consisted of the vowel frames *i-e* and *o-u* while in deviants, the incorrect pairings *i-u* or *o-e* violated the dependency. Consonant standards contained the consonant frames *b-p* and *g-k*, with the pairing violated in deviant items, such that *b-k* and *g-p*.

The stimulus material consisted of trisyllabic sequences of consonant-vowel syllables (CV), individually recorded and spoken by a trained female speaker (see Experiment 2; Weyers & Mueller, 2022). Syllable recordings of similar pitch were selected, digitized (44.1 kHz/16-bit sampling rate, mono), cut to the same length (380 ms) and normalized to the same sound intensity. In the vowel group, standard sequences contained the fixed NAD vowel combinations …i…e and …o…u in the first and last syllable, respectively. The remaining slots in the CVCVCV structure were filled equally often with the consonants */b,g,k,l,m,r,s,w*/ (and the vowels /*a,ä,ö,ü*/ in the middle syllable), avoiding repetitions within items. The fixed consonant frames encoding the NAD in the consonant group were g…k… and b…p…, and the variable positions were filled accordingly with /*d,l,m,s*/ and /*a,e,i,o,u,ä,ö,ü*/ (cf. Fig. 1 for examples). Dependency deviants were created by pairing the respective non-adjacent vowels (consonants) across frames, i.e., …i…**u** and …o…**e** (g…**p**… and b…**k**…), thereby violating the established NAD. For intensity deviants, the sound pressure level of the entire final syllable was reduced by 12 %. This was equal to approximately 10 dB and intentionally larger than the decrement of 6 dB shown to be successfully detected already by neonates in non-words (Tarquinio et al., 1990) or tones (Sambeth et al., 2009), because we wanted to ensure that the intensity violation, which also served as a control condition, was definitely noticeable to the infants. In case of the absence of any significant ERP effects in this condition, we would have to conclude that infants simply did not listen to the input attentively enough for them to detect the acoustic manipulation, let alone the linguistic one.

### 2.3 Procedure

We used a classic oddball paradigm, hence ∼ 72 % of the 448 items used in each experimental group were standards (S, *N* = 320) interspersed with ∼14% of deviants (D, *N* = 64) per violation condition. Note here that for an intensity deviant, a preceding dependency deviant qualifies as a standard and vice versa (Näätänen et al., 2004). The trisyllabic sequences were presented with 50 ms pauses between syllables within a sequence and 700 ms pauses between sequences (see Mueller et al., 2012; Peña et al., 2002). Four different stimulus lists were created per experimental group, in which the sequence of items was pseudo-randomized according to the following constraints: the first 16 items of each list consisted of non-repeating standards for familiarization, a deviant was always preceded by a minimum of four and a maximum of eight standards, and the same type of deviant (dependency or intensity) could appear a maximum of three times in a row.

### 2.4 Data collection and pre-processing

#### 2.4.1 EEG data

Most of the participants were tested at the Kindersprachlabor of the University of Osnabrück, though the first seven participants were tested at the Children’s Laboratory of the Max-Planck-Institute for Human Cognitive and Brain Sciences in Leipzig (MPI-CBS). Set-up and materials were closely matched in both locations: both used a 27 Ag/AgCl electrode cap (EASYCAP, Germany; International 10–20 system of Electrode Placement) with AFz serving as ground, and a TMSi 72 Refa amplifier system (TMSI B.V., Netherlands). At the MPI-CBS, the data were continuously recorded with the QRefa Acquisition Software, Version 1.0 beta (MPI-CBS, Leipzig, Germany) at a sampling rate of 500 Hz with Cz serving as an online reference and a monopolar electrode (EOG) placed below the right eye recording the electrooculogram. At the University of Osnabrück, the data were recorded using the TMSi Polybench recording software (TMSI B.V., Netherlands), sampled at 512 Hz using an average online reference and EOG placed below the left eye. Impedances were kept below 30 kΩ. During the experiment, infants were seated on a caregiver’s lap, while stimuli were played via loudspeakers at a constant sound level across participants.

The EEG data were processed offline with MATLAB (version R2020b, The MathWorks Inc., 2010) and the EEGLAB open source toolbox (version 14.1.1b, Delorme and Makeig, 2004). The datasets recorded in Leipzig at 500 Hz were re-sampled to match the sampling rate of the majority of the datasets, which were recorded at 512 Hz in Osnabrück. Even though up-sampling is achieved by interpolating 12 additional data points from the recorded 500 data points per second, this was deemed only a minor adjustment and unlikely to falsely introduce or conceal any potential effects, particularly because only three of the seven datasets recorded in Leipzig entered the final analysis. The raw data were detrended and, in a semi-automatic procedure, EEGLAB’s built-in kurtosis computation option was used and any channels with values 3 standard deviations from the mean were marked as bad. A maximum of two bad channels were visually selected and interpolated per dataset. If more than two bad channels were identified, the entire data set was excluded from analysis (*N* = 7). The data were then re-referenced to the averaged signal recorded from the mastoids and band-pass filtered twice, using separate digital windowed sinc FIR-filters (window type: Kaiser): once with a 0.3 Hz high-pass filter (− 6 dB half-amplitude cutoff, filter order 3710, transition width of 0.6 Hz) and a 30 Hz low-pass filter (− 6 dB, 188, 10 Hz), and once with a 1 Hz high-pass filter (− 6 dB, 930, 2 Hz) and a 30 Hz low-pass filter (− 6 dB, 188, 10 Hz). Both resulting datasets were epoched in time windows of − 100 to 800 ms. In the consonant condition, zero marks the onset of the final syllable, which equals the onset of the violation (e.g., gido=pu). Since in the vowel condition, the onset of the dependency violation is on the vowel within the final syllable, a cutoff point was defined in Audacity and a trigger placed in between consonant and vowel of the final syllable, marking the onset of the epoch (rowäk=e). For comparison, the same epoch onset was used in the intensity condition of the vowel group, even though the intensity manipulation was applied to the entire final syllable. In each group, all standards appearing immediately after a deviant (dependency or intensity) were removed from analysis to avoid re-familiarization effects. In a first artifact rejection procedure, epochs containing severe artifacts (e.g., due to muscle movements or amplifier saturation) were manually removed from both datasets. Independent component analysis (ICA) was then run on the 1–30 Hz filtered dataset, eye-movement related components were rejected and ICA weights applied to the 0.3–30 Hz filtered dataset. In a second, semi-automatic threshold rejection procedure (setting a threshold of ± 150 µV in EEGLAB), any remaining epochs containing artifacts were rejected (cf. Table 1 for average number of trials per condition and group). Finally, the data were baseline-corrected (− 100 to 0 ms) and condition means per participant and group were created. An additional 10 Hz low-pass filter (− 6 dB, 10, 620) was applied to the averaged data exclusively for plotting in order to improve visibility.

#### 2.4.2 Development test data

In addition to the pre-registered experiment, we conducted the ET 6-6-R development test (Macha and Petermann, 2008, Macha and Petermann, 2013) with all infants at a second appointment with a maximum of seven days delay. Since the age group selected for the present study fell between two age groups defined in the ET 6-6-R, we used the 7.5–9 months version of the test for all 8- to 10-month-old infants in our study for comparability across participants.

### 2.5 Statistical analysis

The EEG data were analyzed statistically using the FieldTrip toolbox for EEG/MEG-analysis (Oostenveld et al., 2011). Separate non-parametric cluster-based permutation tests were run for each experimental group, comparing ERP responses to standards and deviants using dependent samples *t*-tests. EOG and reference electrodes as well as the baseline window (− 100 to 0 ms) were excluded from analysis. A minimum of two significant neighboring electrodes was defined for a significant EEG data sample to be included in a cluster and neighbors were identified with a spatial neighborhood template using the triangulation method. The sample-specific test statistic threshold was set to p < .05. For the cluster-statistic permutation test, we used the maximum sum approach, and significance probabilities were estimated based on 1000 draws from the permutation distribution via Monte-Carlo sampling and an alpha level of p < .05 (distributed over both tails). Additional regression analyses were planned for each group, with mean ERP amplitudes of the intensity condition (intensity deviants minus standards) as predictors and mean ERP amplitudes of the dependency condition (dependency deviants minus standards) as criterion. The time windows for these analyzes were determined by statistically significant condition effects resulting from the previously described analysis.

## 3 Results

### 3.1 Consonant group

**Fig. 2:**
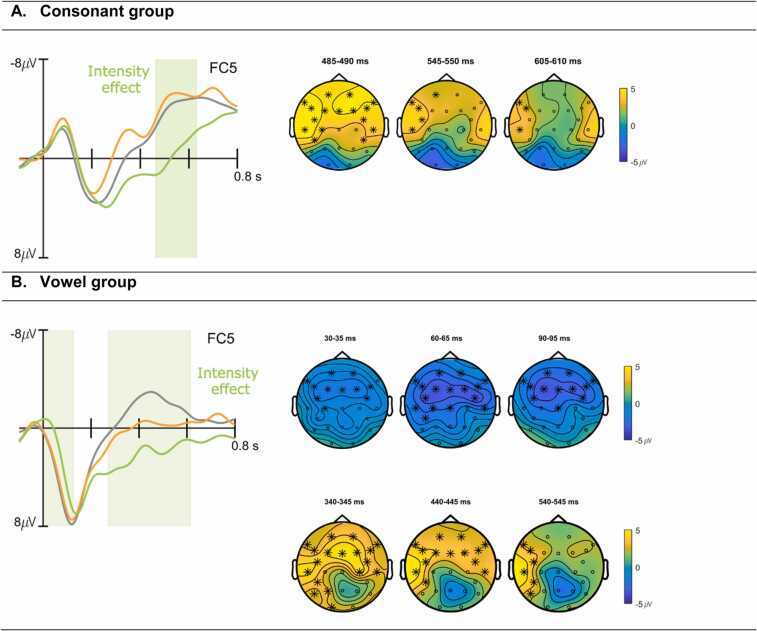
ERP waveforms and topoplots for the consonant (A) and vowel (B) group of data averaged by conditions (standard = grey, intensity deviant = green, dependency deviant = orange) at a representative electrode. Significant differences between the standard and intensity condition are shaded in light green. Topographical plots show 5 ms averaged time windows that best illustrate distribution and time course of the respective effects, electrodes with significant effects contributing to the cluster are marked with asterisks.

The non-parametric cluster-based permutation tests of averaged ERP responses to standards versus intensity deviants revealed a significant condition effect for the intensity manipulation (clusterstat T = 1.7960e + 03, p = .03). The corresponding electrode cluster spans a time window of approximately^1^ 460–630 ms relative to syllable onset and is observed as a positivity with a fronto-central maximum for intensity deviants compared to standards (Fig. 2A). The equivalent comparison of standards and dependency deviants did not yield any statistically significant differences.

### 3.2 Vowel group

The non-parametric cluster-based permutation tests of averaged ERP responses to standards versus intensity deviants also revealed a statistically significant condition effect for the intensity manipulation (clusterstat T = − 2.4223e + 03, p = .02), at two electrode clusters. The first cluster is represented as a fronto-centrally distributed negativity ranging from around 0–125 ms relative to vowel onset, while the second cluster shows as a positivity between approximately 270 ms and 610 ms with a broad to left-lateralized distribution (Fig. 2B). For the comparison of standards and dependency deviants, again no statistically significant differences between conditions emerged.

### 3.3 Regression analysis

Despite of not finding a statistically significant dependency effect for the consonant nor the vowel group, we ran the pre-registered regression analyses to explore the relationship between intensity and NAD processing. The mean amplitude of the intensity effect in the above reported approximate time windows was used as a predictor for the magnitude of the ERP amplitude difference in the dependency condition. Due to the lack of significant dependency effects, the latter was defined in the same time window as the respective intensity effect. Regression-analyses revealed no significant results, neither in the consonant (r^2^ = .05, p > .05) nor in the vowel group (time window positivity: r^2^ = 0.03, p > .05; time window negativity: r^2^ = 0.01, p > .05).

### 3.4 Development test

Average quotients for the Language Development dimension of the ET 6-6 development test (Macha and Petermann, 2008, Macha and Petermann, 2013) were 11.27 (SD = 2.3) in the consonant group (*N* = 33) and 11.51 (SD = 2.28) in the vowel group (*N* = 35). Welch two-sample *t*-tests revealed no significant difference between groups (p > 0.05

### 3.5 Exploratory analysis

**Fig. 3:**
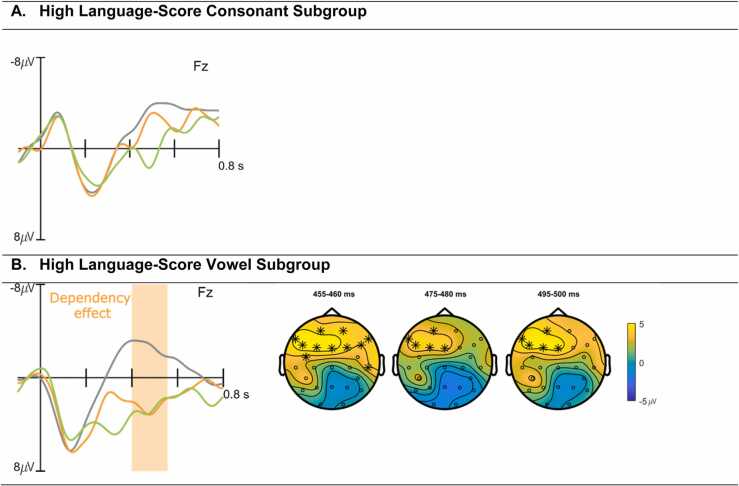
ERP waveforms and topoplots for the consonant (A) and vowel (B) “high language score” subgroups of data averaged by conditions (standard = grey, intensity deviant = green, dependency deviant = orange) at the representative electrodes. Significant differences between standard and dependency condition are shaded in light orange. Topographical plots show 5 ms averaged time windows that best illustrate distribution and time course of the respective effects, electrodes with significant effects contributing to the cluster are marked with asterisks.

In an exploratory analysis not included in the pre-registration, we split the two participant groups based on their performance in the subtest “Language Development” of the ET 6-6-R (Macha and Petermann, 2008, Macha and Petermann, 2013) into a high language-score subgroup (HLS) and low language-score (LLS) subgroup. As the maximum quotient is 14 and the average in the population for the 7.5–9 months version of the test is 10, children with an above average quotient of > 10 were assigned to the HLS subgroup (VOW *N* = 22, CON *N* = 19), and children with a quotient of 10 or lower to the LLS subgroup (VOW *N* = 13, CON *N* = 14). We ran cluster-based permutation tests on the data of the HLS and LLS subgroups for both consonant and vowel groups, comparing ERP responses to standards and dependency deviants. In the vowel HLS group, there was a statistically significant dependency effect (clusterstat T = 1.3354e + 03, p = .034), corresponding to a positivity in an approximate time window of 400–550 ms post violation onset (Fig. 3B), while no such effect was present in the HLS consonant group (Fig. 3A). No significant differences were found in either of the LLS groups. Running the above correlation analysis using the significant time windows of the dependency and intensity effects of the HLS vowel group did not return any significant correlation effects (r^2^ = .002, p > .05).

Due to the fact that we had used the 7.5–9 months version of the development test for our entire age range of 8- to 10-month-olds, we further ensured that language-score was not confounded with age (a possibility raised by an anonymous reviewer). The HLS (*N* = 22, m = 282.64 days, SD = 15.29) and LLS (*N* = 13, m = 277.31, SD = 21.21) vowel sub-groups did not significantly differ with regard to age (as determined by a Welch two-sample *t*-test with p > .05), however, the consonant sub-groups did (p < .05), with the HLS (*N* = 19, m = 288.90, SD = 17.57) being significantly older than the LLS (*N* = 14, m = 271.29, SD = 15.96) group. To further address this point, we performed a median split on age across the whole group (median = 284 days) and again compared the ERP responses to standards and dependency deviants for each age group within each experimental group. There were no significant differences between ERP responses to standards and dependency deviants for any of the four sub-groups tested (all p > .05), suggesting that age was in fact not the relevant factor driving the differences in effects found for the vowel sub-groups.

## 4 Discussion

The present study aimed to investigate whether infants can learn NADs when they are coded at the segmental level, that is, between consonants or between vowels, in comparison to a previous infant study reporting successful learning of item-specific NADs based on syllables (Mueller et al., 2012). We further explored whether individual differences in auditory processing and general language development would show a relation to infants’ NAD learning (Frost et al., 2020, Mueller et al., 2012). In an oddball experiment, we presented two groups of 8- to 10-month-old infants with an artificial language comprising dependencies either between non-adjacent vowels or between non-adjacent consonants. We compared infants’ ERP responses to standards and deviant stimuli, which were either reduced in intensity (intensity deviant) or violated the NAD (dependency deviants). In neither the consonant nor the vowel group did we find ERP evidence of infants’ differential processing of dependency deviants and standards, suggesting that infants had not learned the respective segmental-level NAD. At the same time, both infant groups displayed significantly different ERP responses to intensity deviants compared to standards, indicating successful detection of the acoustic manipulation. In our correlation analyses, we found no evidence for a significant relation between intensity processing and NAD learning in either group. When splitting the groups based on their language development scores, the high-scoring vowel group showed ERP evidence of NAD learning, indexed by a significantly more positive response to dependency deviants compared to standards.

The finding of a null result in the dependency condition, suggesting that 8- to 10-month-old infants did not learn either of the two segmental-level NADs, is unexpected as there is evidence that already 3- to 4-month-olds are able to learn item-specific regularities at the syllabic level (Kabdebon et al., 2015, Mueller et al., 2012). Deviance detection at the segmental level per se should not pose a challenge to infants at this age, since 3-month-olds have been found to already automatically detect both vowel (e.g., Cheour et al., 1998) and consonant changes (e.g., Dehaene-Lambertz and Dehaene, 1994) in simple oddball paradigms. The challenge could have therefore consisted in detecting and encoding the dependency relation between these small segmental units. A potential reason for this challenge may be that by the age of 8–10 months (or earlier), infants have learned to group individual sounds into syllabic units. Similar to adults, who display a clear advantage for syllable-based NAD learning (Weyers and Mueller, 2022), infants at this age may perceive syllables as more salient than individual phonemes. The fact that there was a pause inserted between syllables in the present experiment might have supported syllable instead of phoneme perception. Yet, the pauses between syllables were very short with only 50 ms compared to the inter-stimulus-interval of 700 ms marking the entire trisyllabic unit. Independent of this methodological consideration, earlier work has advocated for the syllable as a natural unit of speech perception already in early infancy: both newborns (Bijeljac-Babic et al., 1993) and 2- to 4-month-olds (Bertoncini and Mehler, 1981, Eimas, 1999) were found to dissociate acoustically manipulated sequences based on syllabic rather than segmental or sound feature representations. Moreover, word segmentation studies suggest that 6- to 8-month-olds are able to group syllables into word-like units using distributional information (e.g., Aslin et al., 1998; Saffran et al., 1996) or prosodic cues (e.g., Cheong and Uehara, 2021; Nishibayashi et al., 2015) and recent modeling work corroborates the importance of syllables in prelinguistic perception and representation from the very beginning (Räsänen et al., 2018). Since infants at around 8–10 months of age also start to produce canonical syllables in their babbling (e.g., Werker and Tees, 1999), it is evident that infants represent syllables as important units of computation at that age.

Yet, there is also evidence that young infants are capable of computing certain phonological relations across non-adjacent vowels and consonants. Specifically, infants learning a language exhibiting vowel harmony (i.e., phenomenon in which vowels within a word are phonologically assimilated, e.g., in their roundness or backness feature) have been shown to successfully discriminate legal vowel-harmonic from illegal (or infrequent) vowel-disharmonic sequences within (pseudo-)words at 10 months (Turkish: Hohenberger et al., 2016, 2017) to 13 months of life (Hungarian: Gonzalez-Gomez et al., 2019). Likewise, infants acquiring French develop a perceptual bias for non-adjacent labial-coronal consonant sequences (e.g., bad, pet, pod), which are frequent in their mother tongue, over less frequent coronal-labial sequences (e.g., dab, top, dop) between 7 and 10 months of age (Gonzalez-Gomez and Nazzi, 2012, Nazzi et al., 2009). Why is it then that infants in the present study were largely unable to identify similar vowel- and consonant-based non-adjacent dependencies? Firstly, it is likely that the abilities reported in the cited studies are based on language-specific knowledge. In fact, Gonzalez-Gomez et al. (2019) showed that in contrast to Hungarian-learning infants, a matched control group of infants acquiring French (i.e., a language that does not typically harmonize) did not exhibit a sensitivity for word-internal vowel harmony. Similarly, our German-learning infants would not be expected to differentiate between standards and deviants based on vowel-harmonic features in the vowel group, nor on the basis of the voiced-voiceless consonant sequences in the consonant group, as these do not constitute a particularly prominent phonotactic pattern in German. Secondly, a methodological difference between the above-cited and the present study could explain the differences in results. Even though all of the above studies intend to investigate *non-adjacent* dependencies, their stimulus materials can arguably be interpreted as consisting of *adjacent* dependencies at the segmental level (cf. Newport and Aslin, 2004, for a similar argument). In Gonzalez-Gomez and Nazzi’s (2012) materials, for instance, the relevant consonants are only separated by one vowel, that is, they are directly adjacent on the consonantal tier (e.g., *b*a*d*, *p*e*t*, *p*o*d*), as are the vowels on the vocalic tier in Gonzalez-Gomez et al.’s (2019) items (e.g., *e*d*ü*, *ö*t*i*, *o*p*a*, *a*d*u*). In contrast, our stimuli comprised intervening elements on the relevant tier, which also resulted in comparatively larger distances between the related segments (e.g., sib**a**we, bä**m**apu). The differences in results could therefore also suggest that segment-based NAD learning is not entirely inaccessible at this age, but rather constrained to a small number of intervening elements. Future research could systematically manipulate this factor and simultaneously test infants from different language backgrounds in order to disentangle these two explanations.

The ERP results from the intensity manipulation confirm our hypothesis that 8- to 10-month-olds would be able to detect syllable-based intensity decrements and therein approve infants’ basic auditory processing abilities at this age. Although there is generally large variability in infants’ auditory ERP components to acoustic novelty and change (compared to adults and older children), the positive-going ERP response we observed aligns with previous findings (Dehaene-Lambertz, 2000, Friederici et al., 2002, Kushnerenko et al., 2002, Kushnerenko et al., 2007, Trainor et al., 2003). Although there is no consensus regarding the functional significance of positive-going as opposed to negative-going discrimination responses (for a review, see Trainor, 2007), they have often been interpreted as either an immature Mismatch Response, which eventually develops into a mature Mismatch Negativity (MMN) (Friederici et al., 2002, Mueller et al., 2012), or resembling an adult P3a-like component, reflecting involuntary orienting of attention and automatic novelty detection (Kushnerenko et al., 2002, Trainor et al., 2001). Independent of the interpretation of the observed intensity effect as positive MMR or P3a-like component, it indicates that infants have successfully detected the intensity manipulation. In addition to the positivity found for both groups, the vowel group also exhibited an early negativity in response to intensity deviants, which has the characteristics of a typical auditory N1 effect. The fact that we do not find this effect in response to intensity deviants across both groups is somewhat surprising, because it is known to be related to acoustic change (e.g., Escera et al., 1998). This difference between groups is somewhat difficult to explain, but we would speculate that it may be a result of the different number and distribution of vowels and consonants in the stimulus materials of the two groups. Our exploration of infants’ intensity processing as a potential predictor for NAD learning, similar to pitch as reported by Mueller et al. (2012) for both adults and infants, did not yield any significant correlations in either infant group. The lack of such a predictive relationship can be explained firstly by a methodological caveat: since we did not find any significant dependency effects across either infant group as a whole and therefore lacked a time window of interest for the dependency condition, we entered the ERP amplitude differences in the time window of the intensity effect into the correlation analysis for both conditions. This choice of course precludes any potential correlation effects outside this relatively arbitrary time window. Our exploratory correlation analysis of the vowel subgroup ERP amplitudes, for which we did find significant ERP effects in the dependency condition, did not yield any significant correlation effects either, however. Any conclusions drawn from these null results would be tentative at best, particularly since NAD learning effects in our experiment were limited overall. We thus suggest that future research further explore the link between NAD learning and auditory perceptual abilities as proposed by Mueller et al. (2012). More specifically, it may be interesting to differentiate between different auditory features, such as pitch and intensity, their relative importance for language and their potentially related predictive value for language processing abilities.

In addition to individual differences in basic auditory processing, we investigated potential effects of individual differences in general language capacity for NAD learning. Interestingly, only those infants who displayed overall advanced language skills and who were exposed to the vowel-based NAD showed ERP evidence of learning, indexed by a significantly more positive response to dependency deviants compared to standards. The fact that only the high language performer group showed evidence of vowel-coded NAD learning may be explained by the properties of the discrimination response itself, which is very similar in timing and distribution to the previously described response to the intensity condition. Typically, discrimination responses like the MMN or P3 increase in amplitude as a function of magnitude of change in the relevant stimuli and have been found to additionally depend on individual differences in arousal and attention (for reviews, see Fitzgerald and Todd, 2020; Polich, 2007). The same stimulus change might hence remain unnoticed by some infants, while others might perceive it as deviant, resulting in a discrimination response (for a similar argument cf. Kushnerenko et al., 2002). In other words, those infants who display stronger early language skills (both receptive and productive) might be more attentive to language input in general and able to pick up even subtle segmental-level regularities and their violation.

Why is it, however, that only the high language performers in the vowel group and not those in the consonant group learned the respective dependency? This result at least partially confirms our initial hypothesis that infants would (overall) show evidence of NAD learning for vowel-based dependencies and only to a lesser degree for consonant-based dependencies. We may see here an effect related to the more general property of vowels as carriers of prosodic information and thereby supporting structural operations in sequence learning (e.g., Bion et al., 2011; Christophe et al., 2003). While this function of vowels has been shown for repetition-based sequences (Hochmann et al., 2011, Pons and Toro, 2010) our study might be taken to extend those findings to item-specific relations spanning intervening material. In contrast, consonants do not seem to lend themselves well to the computation of item-specific NADs even in those infants displaying advanced overall language skills.

Interestingly, the same paradigm with adults yielded (besides a strong syllable advantage for NAD learning) an electrophysiological (but no behavioral) indication of learning based on consonants, but not vowels (Weyers and Mueller, 2022), even though an advantage for vowels in structural processes has been argued to be present in adults as well (e.g., Bonatti et al., 2005). One reason could be that adults already have extensive experience with possible words and grammatical relations in their native (and non-native) language(s). They might know from experience that trisyllabic units typically constitute word units rather than sentences, and that NADs typically involve dependencies between elements longer than one phoneme (although there are exceptions, e.g., in person agreement in German). Therefore, adults may have treated the stimuli as words and their consonantal bias (C-bias) for word identification (e.g., Havy et al., 2014) may have induced automatic lexical learning processes on the consonant-based NADs. Infants lack extensive native language experience to some degree and might thus not have treated the trisyllabic sequences as word units. Overall, infants may have been influenced less by linguistic knowledge and more by general perceptual properties of vowels, which make them good candidates for being memorized (Benavides-Varela et al., 2012).

A related explanation for why German-speaking adults but not infants show evidence of learning consonant-based NADs may lie in the onset of the C-bias in lexical processes. Even if infants in our study treated the trisyllabic units as words like adults, they may not display by a C-bias (yet). The switch from a preference for vocalic information to consonantal information in lexical identification has been reported in Italian and French-learning infants starting from 8 to 11 months of age (e.g., Von Holzen et al., 2018; Von Holzen and Nazzi, 2020). Our results could point to a later switch towards a C-bias in German-learning infants, that is a potential prolonged V-bias for this language. There is no systematic data on V- and C-biases for German yet, and evidence from other Germanic languages, such as Dutch (Swingley, 2005) and English (e.g., Mani and Plunkett, 2007) is mixed, with no consistent pattern imminent yet (for a review, cf. Nazzi and Cutler, 2019). As German is, unlike French and Italian, a stress-timed language, it is well conceivable that segmental units are treated differently across development. A longer preference for vowels could help infants learn the specific and sometimes variable stress patterns, which in German follow a default trochaic pattern (e.g., **Blu**menkohl – ***cau**liflower*) but also include exceptions (e.g., Kar**tof**fel – *po**ta**to*).

## 5 Conclusion

Our ERP study was the first to test item-specific NAD learning at the segmental level in infants. Our findings suggest that similarly as for adults, learning item-specific dependencies between consonants or vowels is relatively challenging for 8- to 10-months-old infants. Our null results for such learning across infant groups could on the one hand indicate that single phonemes may play a lesser role in infants’ sequential dependency learning at this age and that infants may instead rely more on larger units, such as syllables, as, for instance, shown by Mueller et al. (2012) and Kabdebon et al. (2015). On the other hand, these results could imply that segment-based non-adjacent dependency learning at this age is limited to a certain number of intervening elements. Interestingly, infants who displayed advanced overall language skills did show evidence of learning exclusively vowel-based NADs. Thus, it seems that the vowel-advantage postulated for (non-adjacent) repetition-rules extends to non-repetition, item-specific contexts. Further studies may systematically vary consonants, vowels, and constituent sizes to tackle the role of different types and lengths of computational units in sequence learning in more detail.

## Declaration of Competing Interest

The authors declare that they have no known competing financial interests or personal relationships that could have appeared to influence the work reported in this paper.

## Data Availability

Ethics approval and participant consent for data sharing were not obtained. Raw data is not available for sharing.

## Footnotes

1 It is important to note that the cluster T-statistic obtained in a cluster-based permutation test does not allow for the determination of the specific spatiotemporal characteristics of the identified clusters. That is because for individual sample statistics to be included in a cluster, there is no error rate control. The temporal and spatial distributions of the identified effects are, however, most likely highly correlated with those of the true effect (Groppe et al., 2011).
